# Rupture spontané de la rate: à propos d’un cas et revue de la littérature

**DOI:** 10.11604/pamj.2017.27.62.12451

**Published:** 2017-05-29

**Authors:** Safae El Abbadi, Fatima Zahra Rhouni, Laila Jroundi

**Affiliations:** 1 Service de Radiologie des Urgences, CHU-Ibn Sina, Université Med V, Rabat, Maroc

**Keywords:** Hématome sous capsulaire splénique, non traumatique, hémopéritoine, Subcapsular splenic hematoma, non-traumatic, hemoperitoneum

## Abstract

Ce travail rapporte le cas d’un hématome sous capsulaire spontané rompu de la rate (avec hémopéritoine) et fait le point sur cette pathologie rare. Les ruptures non traumatiques peuvent être mortelles, le diagnostic est parfois difficile. Elles révèlent souvent une pathologie sous-jacente, notamment infectieuse, tumorale ou hématologique. La symptomatologie est habituellement aiguë, mais des formes progressives sont possibles. La majorité des patientssont splénectomisés.

## Abstract (en)

This study reports a case of spontaneous subcapsular splenic ruptured hematoma (with hemoperitoneum) and provides update on this rare disease. Non-traumatic ruptures can be fatal. Diagnosis is sometimes difficult. They often reveal an underlying pathology including infections, tumors or hematologic disorders. Symptomatology commonly has an abrupt onset but progressive forms are also possible. The majority of patients undergo splenectomy.

## Introduction

Les hématomes spontanés de la rate sont rares mais peuvent être mortels [1]. La mortalité est souvent liée au retard diagnostique et de la prise en charge thérapeutique, ainsi qu´aux risques liés à la gravité de la pathologie sous-jacente [2,3]. Elles peuvent survenir sur une rate normale ou pathologique. Le traitement consiste souvent en une splénectomie. Certains malades ayant une MNI peuvent bénéficier d’un traitement conservateur.

## Patient et observation

Il s’agit d’un homme de 40 ans sans antécédents particuliers adressé aux urgences pour des douleurs abdominales diffuses d´installation brutale lors de son sommeil.Le patient était pâle, apyrétique et avait une défense abdominale. Le bilan biologique était normal. L´ASP montrait un iléus diffus.

**Figure 1:**
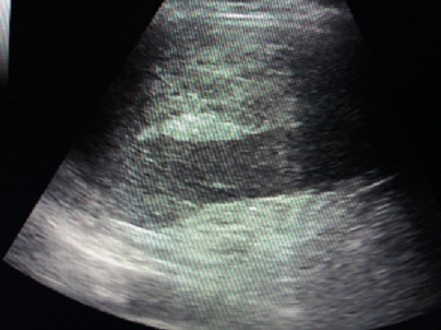
Echographie montrant une plage échogène de la loge splénique

**Figure 2:**
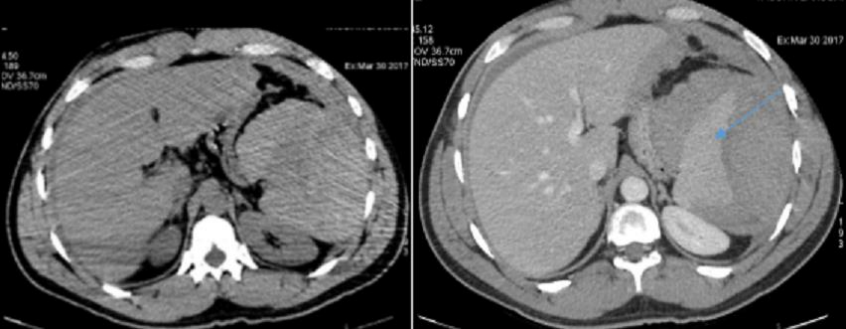
Scanner abdominale C-C+ en coupe axiale: hématome sous capsulaire splénique rompu et hémopéritoine

**Figure 3:**
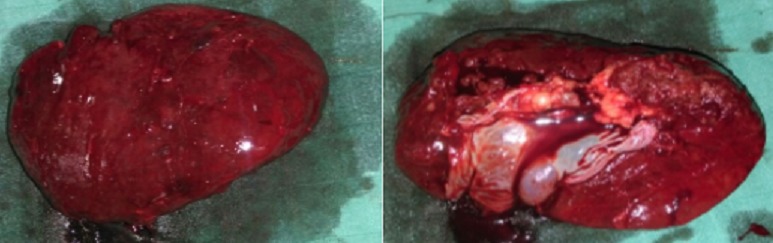
Rate macroscopiquement saine

L’échographie abdominale a montré une plage échogène hétérogène de la loge splénique (Figure 1), un épanchement péritonéal périhépatique, dans les gouttières pariétocoliques et le CDS de Douglas. Le scanner abdominal évoquait le diagnostic de rupture de rate en confirmant l´existence d´un hémopéritoine de moyenne abondance et un hématome sous capsulaire splénique rompu (Figure 2). Lors de la laparotomie, il existait un hémopéritoine en rapport avec une décapsulation complète d´une rate d´aspect sain (Figure 3). Une splénectomie était réalisée. L´examen histologique a confirmé l´aspect non pathologique d´une rate décapsulée.

L’enquête étiologique chez notre patient s’est avérée négative. Il n’existait aucun signe clinique ou paraclinique en faveur d’une cirrhose. L’absence de syndrome clinique et biologique infectieux ou encore de voyage à l’étranger rendait peu probable l’origine infectieuse. Une maladie systémique ou une hémopathie ont a priori été éliminées.

## Discussion

Les ruptures non traumatiques de la rate (RNTR) peuvent se produire chez 0,1% à 0,5% des patients sans Traumatisme associé [4]. Les premiers cas de rupture splénique spontané ont été décrits par Rokitansky [5] en 1861 Et Atkinson [6] en 1874. La cause réelle de la rupture n´est pas encore bien identifiée. Trois mécanismes Ont été impliqués dans le processus: l’augmentation de la tension intrasplénique liée à l’hyperplasie cellulaire et à l’engorgement; la compression par la musculature abdominale lors des efforts d’éternuement, de toux ou de défécation; l’occlusion vasculaire par l’hyperplasie du réticulum endothélial responsable d’infarcissement associée ou non à un hématome sous-capsulaire [7]. Les RNTR sont deux fois plus fréquentes chez les hommes. L´âge varie de 2 à 81 ans (moyenne = 42 ans). Il existe dans environ un tiers des cas des signes de choc lors du premier examen. Dans 8% des cas, les malades décèdent avant d´être opérés et le diagnostic n´est fait qu´à l’autopsie [8].

Les causes de RNTR [8] sont dominées par les maladies infectieuses et hématologiques qui représentent plus de la moitié des cas. Les causes infectieuses (30 %) sont généralement représentées par la MNI et le paludisme, tandis que les causes hématologiques (27 %) sont surtout représentées par les hémopathies malignes. D´autres causes sont beaucoup plus rares: tumeurs solides ou bénignes de la rate (11 %), les pathologies digestives (pancréatite, hypertension portale) (10 %), les causes rhumatologiques (4 %) et l´insuffisance rénale au stade de dialyse (3 %). Dans près de 5 % des cas, aucune étiologie et aucune notion de traumatisme ne sont retrouvées comme le cas de notre observation [8].

Les RNTR sont souvent révélées par un tableau d’urgence chirurgicale. Il peut s’agir d’une forme aiguë réalisant un état de choc hypovolémique, ou subaiguë se présentant sous forme d’une douleur abdominale diffuse prédominanteà gauche, associée à une hypotension et à une anémie [9, 10]. Ces troubles circulatoires sont dus à la spoliation sanguine qu’est l’hémopéritoine. Cet hémopéritoine, survenant en dehors d’un traumatisme, pose un problème du diagnostic étiologique. L’existence d’une douleur abdominale et d’une splénomégalie massive douloureuse, oriente vers une atteinte splénique devant être confirmée en urgence par l’échographie qui est l’examen de première intention. Cependant, la tomodensitométrie présente une meilleure sensibilité pour faire le bilan lésionnel [11].

Sur le plan thérapeutique, la splénectomie est le traitement radical des ruptures spontanées de la rate. Néanmoins, la morbidité de la splénectomie, l’amélioration des techniques chirurgicales et des soins intensifs ainsi que le rôle de la rate dans la réponse immunitaire nous autorisent à proposer un traitement conservateur. Celui-ci semble être une alternative sous réserve de certaines conditions: stabilité hémodynamique, recours à la transfusion de moins de 2 culots érythrocytaires, surveillance clinique quotidienne et biologique régulière, repos et hospitalisation dans un service proche d’un centre chirurgical [12].

## Conclusion

La rupture spontanée de la rate est une entité rare dont le diagnostic est difficile en absence de contexte traumatique. Elle peut engager le pronostic vital du patient. L'échographie et le scanner permettent d’orienter le diagnostic. Le traitement est essentiellement chirurgical par splénectomie.

## Conflits d’intérêts

Les auteurs ne déclarent aucun conflit d'intérêt.

## References

[1] Lippstone MB, Sekula-Perlman A, Tobin J, Callery RT (1998). Spontaneous splenic rupture and infectious mononucleosis in a forensic setting. Del Med J..

[2] Debnath D, Valerio D (2002). A traumatic rupture of the spleen in adults. J R Coll Surg Edinb..

[3] Schwarz M, Zaidenstein L, Freud E, Neuman M, Ziv N, Kornreich L, Zer M (1999). Spontaneous splenic rupture in infectious mononucleosis: conservative management with gradual percutaneous drainage of a subcapsular hematoma. Pediatr Surg Int..

[4] Lai PK (1977). Infectious mononucleosis: recognition and management. Hosp Pract..

[5] Laseter T, McReynolds T (2004). Spontaneous splenic rupture. Mil Med..

[6] Badenoch DF, Maurice HD, Gilmore OJ (1985). Spontaneous rupture of a normal spleen. J R Coll Surg Edinb..

[7] Mokashi AJ, Shirahatti RG, Prabhu SK, Vagholkar KR (1992). Pathological rupture of malarial spleen. J Postgrad Med..

[8] Kianmanesh R, Aguirre HI, Enjaumeb F, Valverdec A, Brugièred O, Vacher B, Bleichner G (2003). Ruptures non traumatiques de la rate: trois nouveaux cas et revue de la littérature. Annales de Chirurgie..

[9] Bauer TW, Haskins GE, Armitage JO (1981). Splenic rupture in patients with hematologic malignancies. Am Cancer Soc..

[10] Rhee SJ, Sheena Y, Imber C (2008). Spontaneous rupture of the spleen: a rare but important differential of an acute abdomen. Am J Emerg Med..

[11] Delgado Millan MA, Deballon PO (2001). Computed tomography, angiography and endoscopic retrograde cholangiopancreatography in the nonoperative management of hepatic and splenic trauma. World J Surg..

[12] Papp C, Debord T, Imbert P, Lambotte O, Roué R (2002). Rupture de rate au cours des maladies infectieuses: splénectomie ou traitement conservateur? Àpropos de trois cas. Rev Med Interne..

